# Liver Abscess Formation Following Transarterial Chemoembolization: Clinical Features, Risk Factors, Bacteria Spectrum, and Percutaneous Catheter Drainage

**DOI:** 10.1097/MD.0000000000003503

**Published:** 2016-04-29

**Authors:** Wei-Fu Lv, Dong Lu, Yu-Sheng He, Jing-Kun Xiao, Chun-Ze Zhou, De-Lei Cheng

**Affiliations:** From the Department of Radiology, Affiliated Anhui Provincial Hospital of Anhui Medical University, Hefei, China.

## Abstract

To investigate the clinical features, risk factors, and bacterial spectrum of liver abscess following transarterial chemoembolization (TACE) and evaluate the therapeutic effect of percutaneous catheter drainage (PCD) on the abscesses.

A retrospective review of patient charts was performed in 3613 patients who suffered from liver malignancies (2832 patients with hepatocellular carcinoma and 781 with metastatic hepatic tumor) and had undergone 11,054 TACE procedures from January 2005 to October 2013. Liver abscesses were found in 21 patients. PCD was performed in all abscess patients. The clinical features, risk factors, and bacterial spectrum of liver abscess following TACE were investigated and the therapeutic effect of PCD was evaluated.

The incidence of liver abscess was 0.58% per patient and 0.19% per procedure. Approximately 57.1% of the patients had a medical history of bilioenteric anastomosis or biliary stent implantation. On computed tomography scans, the abscesses appeared as low-attenuation lesions and high-density iodinate oil scattered in the abscesses. The ultrasound showed the well defined, heterogeneously hypoechoic lesions. Positive microbiological isolates were obtained in all pus cultures and in 47.6% of blood cultures. The most common bacterium was *Escherichia coli* (52.4%). Twenty patients (95.2%) were cured from abscesses by using PCD, and 1 died of sepsis.

Patients with predisposing factors are prone to an increased risk of liver abscess following TACE. Bacterial culture and antibiotic sensitivity tests on pus and blood help on the antibiotics selection. PCD combined with aggressive antibiotics can be recommended as the first-line therapeutic regimen.

## INTRODUCTION

Transarterial chemoembolization (TACE) is an effective treatment method for unresectable hepatocellular carcinoma (HCC) and metastatic hepatic tumor (MHT). Despite its minimum invasion and significant therapeutic effect, TACE can cause severe complications relative to conservative management.^1–4^ Liver abscess formation is a severe complication of this method.^3,4^ The reported mortality rates due to TACE complications range from 13.3% to 50%.^5,6^

Risk factors that have been reported to be associated with liver abscess formation following TACE include bilioenteric anastomosis, biliary abnormalities, old age, diabetes mellitus, large tumor size, and portal vein occlusion.^3–6^ Meanwhile, the therapeutic strategies for liver abscess following TACE are different from primary liver abscess because of individual patients’ critical pathophysiological status, such as advanced tumors, hepatic dysfunction, ascites, and dyscrasia. Percutaneous catheter drainage (PCD) is recommended as an effective method for liver abscess.^4,5^ However, insufficient information is available about the therapeutic effect because of the small sample size used in previous studies.^5,6^ Furthermore, the investigation of bacterial spectrum is vital because of its guiding value in antibiotics selection. The purpose of this study is to investigate the clinical features, risk factors, and bacterial spectrum of liver abscess following TACE and to evaluate the therapeutic effect of PCD.

## MATERIALS AND METHODS

### Patients

This study was approved by our institution's ethical committee and abided with the Helsinki Declaration. A retrospective review of electronic medical records was performed among 3613 patients who suffered from hepatic malignancies (including 2832 HCC patients and 781 MHT patients) and had undergone 11,054 TACE procedures in the Departments of Interventional Radiology at our institution from January 2005 to October 2013. Among the 3613 patients, 2384 were males and 1229 were females. Their ages ranged from 32 to 76 years, with a mean average of 52.3 ± 21.6 years. Among the 2832 HCC patients, 973 were confirmed by puncture biopsy or surgical-histopathological examinations; the remaining 1859 were diagnosed in accordance with the recommendations of the American Association for the Study of Liver Diseases. MHT diagnosis was determined by at least 2 imaging modalities of ultrasonography, computed tomography (CT), and magnetic resonance imaging and the primary tumors were histopathologically proven. A total of 21 liver abscesses were identified. Fungal or amebic abscesses, infected tumors, or infected liver cysts were excluded from this study.

The TACE indications for hepatic malignancies were based on the following criteria: unresectable hepatic tumors because of either advanced stage or insufficient hepatic reserve; tumors unsuitable for other local treatments such as radiofrequency ablation or percutaneous ethanol injection because of tumor size, multiple lesions, vascular invasion or sub-capsular lesions; an Eastern Cooperative Oncology Group (ECOG) performance status score of 0 to 2; and 18 years of age or older. The exclusion criteria for TACE included the following: any contraindication to the use of arterial procedures, such as impaired clotting (platelet count <50 × 10^9^ per L or prothrombin activity <50%), bacterial infection, or renal failure (creatinine >2 mg/dL), and lesions occupying more than 75% of the whole liver. The baseline characteristics of the patients are listed in Table 1.

**TABLE 1:**
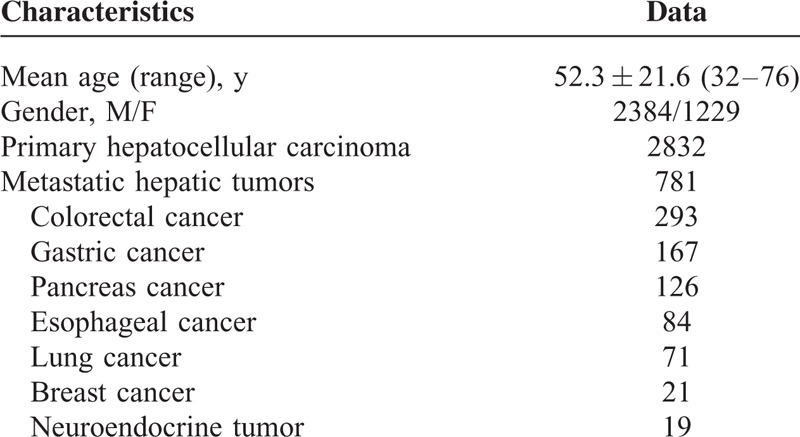
Patient Baseline Characteristics (n = 3613)

### TACE Methods

TACE was performed by the senior interventional radiologists with more than 10 years clinical experience using the similar methods described by other authors.^1,4,7^ The patients were given local anesthesia with 5 mL of 2% Lidocaine (Shanghai No. 1 Biochemical Pharmaceutical Co., Ltd., Shanghai, China). The puncture of right common femoral artery was performed with a 5.0 Fr micro-puncture introducer set (Terumo, Tokyo, Japan) by using the Seldinger technique. The celiac trunk was catheterized with a 4.0 Fr or 5.0 Fr RH catheter (Cook, Bloomington, IN), and the angiographies were completed. Then, the catheter was introduced to the hepatic arteries and advanced further into segmental arteries by using selective catheterization. The micro-catheter was applied if necessary. A mixture of 5 to 20 mL of 40% lipiodol (Andre Guerbet, Aulnay-sous-Bois, France) and 10 mg/m^2^ mitomycin C (Medac, Hamburg, Germany) was slowly injected into the feeding arteries through the catheter until the tumor-feeding branches were near stasis. The other anticancer drugs (determined by the cellular type of primary tumor and body surface area) were infused through the catheter afterward. Finally, the 350 to 510 micron polyvinyl alcohol particles (Cook) were used to embolize the feeding arteries.

### Observation After TACE and Diagnosis of Liver Abscess

After TACE, the patients were closely observed for TACE-related complications. Liver CT scans were performed on patients with suspected liver abscess by applying a 64-row multidetector CT scanner (Lightspeed 64-slice VCT; GE Medical Systems Ltd., Milwaukee, WI) in cinescan mode, with volume coverage from the diaphragm to the iliac crest, continuous scanning, 5 mm slice thickness, tube voltages of 100 to 120 kV, tube currents of 150 to 300 mAs/Auto mAs, 1 s per 360° revolution, and a matrix size of 512 × 512 pixels. If a contrast enhanced CT is necessary, the triple-phase scans (arterial, portal venous, and equilibrium phases) were performed after an intravenous bolus injection of nonionic iodinated contrast material (Omnipaque, GE Healthcare; with a dose of 1.5 mL/kg body weight) at a rate of 4.5 mL/s via a 20-gauge intravenous catheter in the antecubital fossa. CT images were analyzed in consensus by 2 experienced radiologists. Meanwhile, blood and pus cultures and antibiotics sensitivity tests were performed. Liver abscess was diagnosed in CT images showing typical low-attenuation lesions with or without air–fluid levels. Moreover, any 1 of the following conditions was included: blood or aspirate culture revealed bacteria; aspirate from percutaneous drainage had a purulent appearance; and temperature higher than 38.5°C lasted for more than 5 days with leukocyte count >12 × 10^9^ per L without other causes.^8^ When the diagnosis of liver abscess was confirmed, broad-spectrum antibiotics were empirically administered. The most commonly used antimicrobial regimens were 3rd-generation cephalosporin and fluoroquinolones.

### PCD Methods

**FIGURE 1:**
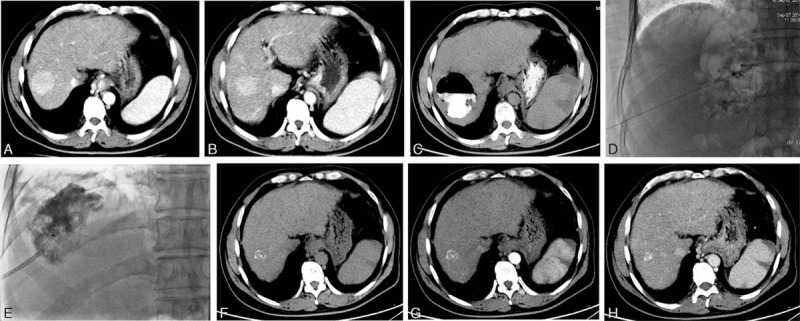
PCD of liver abscess following TACE and CT features of abscess absorption. A 44-y-old male patient presented with liver abscess 12 d after TACE because of hepatic metastasis 50 d after resection of pancreatic cancer. The enhanced CT scans before TACE show a hypervascular lesion within the segment VIII of the liver on arterial phase (A) and portal venous phase (B). On the 23rd d post-TACE day, plain CT scans reveal the gas–liquid level formation in the lesion, and the high-density iodinate oil is disclosed (C). During PCD, the abscess is punctured by using a 21-gauge needle under the guidance of fluoroscopy (D); a 12-Fr drainage catheter is placed inside the abscess cavity (E). On the 42nd post-PCD day, plain (F) and enhanced (G: arterial phase; H: portal venous phase) CT scans show that the liver abscess has completely resolved. CT = computed tomography, PCD = percutaneous catheter drainage, TACE = transarterial chemoembolization.

PCD was performed on all 21 patients. The drainage procedures were listed as follows: CT manifestations of abscesses were reviewed for planning the suitable puncture site; sterile preparation for the skin, draping, and anesthesia of the puncture site with 2% Lidocaine was performed; abscess puncture under the guidance of ultrasonography, CT, or digital subtraction angiography was first given using a percutaneous access set (21-gauge needle; Cook); when the needle penetrated the abscess, a 0.018-inch (1 inch = 2.54 cm) guide wire was inserted into the abscess cavity through the needle, and then a 6-Fr puncture introducer was inserted in place of the needle though the guide wire; the dilator was pulled out from the introducer, and 3 to 5 mL of pus was aspirated for the bacterial culture and antibiotic sensitivity tests; a 0.038-inch guide wire (Terumo) was inserted into the introducer, and a 12-Fr drainage catheter (Cook) was placed in the abscess cavity through the guide wire after pulling out the introducer (Figure 1A–E); and radical pus aspiration and abscess cavity irrigation with 0.9% saline were performed.

### Post-PCD Management

After PCD, broad-spectrum antibiotics were intravenously given until the results of bacterial culture and antibiotic sensitivity tests were elucidated. Afterward, sensitive antibiotics were applied in accordance with the sensitivity test results. The abscess cavity was irrigated once a day for 3 to 5 days after PCD. The patients were closely monitored using either abdominal CT scans or ultrasonography every 7 to 10 days until the abscesses were cured or became <2 cm in size. Finally, the drainage catheter was pulled out.

### Statistical Analysis

The medical records of the patients were investigated with respect to clinical features, laboratory data, number and size of tumor and abscess, associated diseases, bacteriology, drained pus volumes, drainage duration, therapeutic outcome, and complications. All continuous data were expressed as mean ± standard deviations, and categorical variables were reported as percentages. Comparison of qualitative data was determined by Fisher exact test or χ^2^ test. Differences were considered statistically significant at *P* < 0.05. Statistical analyses were conducted using SPSS 17.0 for Windows (SPSS Inc., Chicago, IL).

## RESULTS

### Clinical Features

Liver abscesses were diagnosed within 11 to 23 days (mean, 12.5 ± 7.8 days) after TACE, and the incidences of liver abscess following TACE were calculated in 0.58% (21/3613) per patient and 0.19% (21/11,054) per procedure. Among the 21 patients, 15 were males and 6 were females, ranging in age from 41 to 73 years (mean 54.6 ± 19.1 years). Nine of these patients had HCC, and 12 had MHT. A higher incidence of liver abscess was found in MHT patients than in HCC patients (1.54% vs 0.32%, *P* < 0.01). Among these abscess patients, 16 (76.2%) presented with upper abdominal pain, 13 (61.9%) experienced a high-grade fever, and 9 (42.9%) had vomiting and nausea. During the physical examination, 11 (52.4%) experienced tenderness in their right upper abdomen, and 8 (38.1%) had localized guarding.

The Child–Pugh classes were A, B, and C in 11 (52.4%), 8 (38.1%), and 2 patients (9.5%), respectively. The widest tumor diameter ranged from 3.7 to 9.6 cm (mean, 6.5 ± 3.2 cm). A total of 12 patients (57.1%) had a medical history of either bilioenteric anastomosis (in 11) or biliary stent implantation (in 1). Hypoalbuminemia, portal vein tumor embolus, and diabetes mellitus were complicated in 10 (47.6%), 6 (28.6%), and 5 (23.8%) patients, respectively. Nine patients (42.9%) underwent particulate embolization.

The difference of risk factors for liver abscess formation following TACE was compared between HCC and MHT patients. The incidence of bilioenteric anastomosis was significantly higher in HMT patients than in HCC patients (*P* < 0.01). However, the incidences of hypoalbuminemia and portal vein tumor embolus were significantly higher in HCC patients than in HMT patients (*P* < 0.05) (Table 2).

**TABLE 2:**
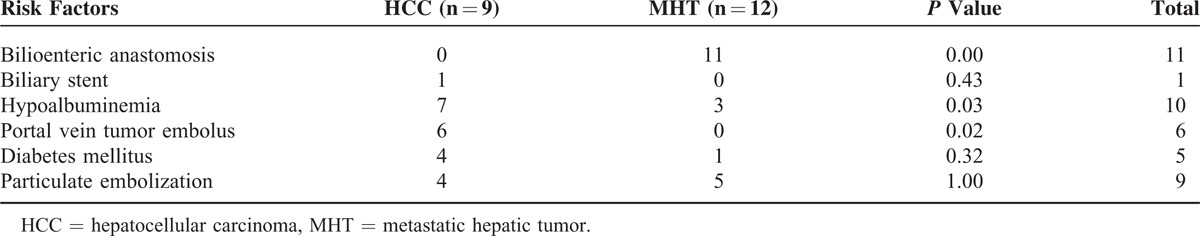
Comparison of Risk Factors Between Patients With HCC and Those With MHT

### CT and Ultrasonography Features

CT scans revealed low-attenuation lesions with thick walls, which were located inside the embolized tumors. Meanwhile, high-density iodinate oil was observed inside the abscesses, and enhanced CT scans could not provide more information for the abscess diagnosis because of the presence of iodinate oil. These findings may be the typical features of liver abscess following TACE. The abdominal ultrasound showed the well defined, heterogeneously hypoechoic lesions. Fourteen patients (66.7%, 14/21) had single abscess, and 7 (33.3%, 7/21) had multi-abscesses. Therefore, a total of 35 abscesses were identified. The widest diameter ranged from 5.7 to 14.9 cm (mean, 8.7 ± 3.2 cm). The gas-formation and air–fluid levels were observed in 8 abscesses (Figure 1C).

### Microbiology

Positive pus cultures were obtained in all 21 patients. Among these patients, mono-microbial culture was observed in 14 patients (66.7%), 2 organisms in 4 patients (19.0%), and 3 organisms in 3 patients (14.3%). Therefore, a total of 31 organisms were isolated. Meanwhile, 12 organisms were isolated from the blood culture of 10 patients (47.6%). In this study, the most common bacterium was *Escherichia coli* (52.4%, 11/21), followed by *Enterobacter cloacae* (38.1%, 8/21), and then *Enterococcus faecalis* (28.6%, 6/21). No significant difference in bacterial spectrum was detected between HCC and MHT patients (*P* < 0.05). Table 3 shows the organisms identified from the abscess and blood cultures.

**TABLE 3:**
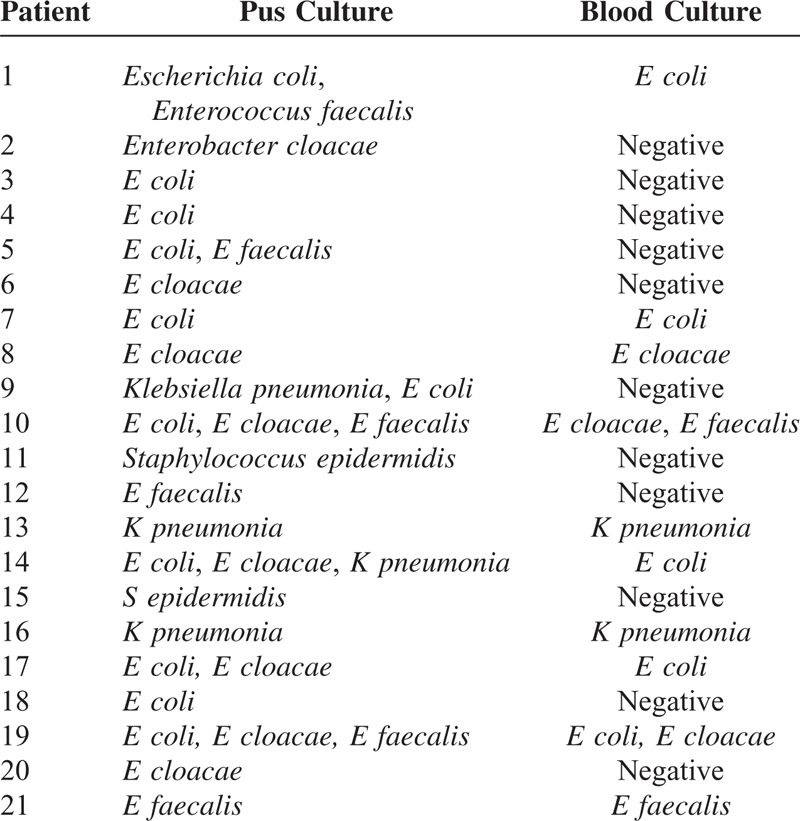
Organisms Identified From the Pus and Blood Cultures

### Drainage Outcome and Follow-Up

The total volumes of drained pus ranged from 78 to 925 mL (average, 464.3 ± 158.6 mL). Three patients needed 2 drainages, and 2 needed 3 drainages. The drainage catheters were left in place for 6 to 16 days (average, 12.2 ± 3.6 days) and were removed only when the abscess cavity had already collapsed around the catheter or had become <2 cm in size. A total of 20 patients were completely cured from abscesses (with a 95.2% curative rate) 15 to 31 days after PCD (average, 18.1 ± 6.3 day) (Figure 1F–H), but 1 patient died of severe sepsis 9 days after PCD (with a 4.8% mortality rate). After discharge, all patients were followed up for at least 3 months. No abscess recurrence was observed in all of these patients.

### Complications

Two patients (12.5%) developed complications following PCD: 1 had abscess cavity-bile duct fistula, and the other had localized peritonitis. The latter was caused by the displacement of the drainage catheter.

## DISCUSSION

In this study, liver abscesses were found in 21 out of 3613 patients with hepatic malignancies who underwent 11,054 TACE procedures, with a 0.58% incidence of liver abscess per patient and 0.19% per procedure. This result agrees with a previous study by Song et al,^5^ who reviewed the charts of TACE in 2459 Korean patients with hepatic tumors and revealed a liver abscess incidence of 0.2% per patient. By contrast, Kim et al^9^ conducted a retrospective analysis of 397 procedures in 157 patients in the University of Pennsylvania's Health System and found liver abscess occurring in 4.5% of patients and 2% of procedures. Their results appear to be higher than ours. The incidence of liver abscess following TACE is statistically discrepant among different institutions and regions, which is likely attributed to the heterogeneous populations, variation in chemoembolization treatment, and numbers of patients studied.^10^

In the present study, the most common symptom of liver abscess is upper abdominal pain (76.2%), followed by high-grade fever (61.9%). These symptoms are nonspecific and are partially similar to those of postembolization syndrome, which are characterized by fever, nausea, vomiting, abdominal pain, and leukocytosis.^10,11^ However, the time course can generally be used to differentiate the 2. Symptoms of postembolization syndrome typically abate by 1 week, whereas presentation of hepatic abscess is usually delayed. Liver abscess should be considered in any patient with suggestive symptoms beyond 7 days.^10^

CT scans have high sensitivity and accuracy in diagnosing liver abscess. This technique can also expose the abscess conditions, such as size, number, location, liquefaction, fibrous septa, and biliary pathology.^12^ In the present study, the majorities of liver abscesses (66.7%) comprised solitary lesion, whereas the minorities (33.3%) were multiple lesions. The abscesses in CT scans were of lower attenuation relative to the surrounding normal liver parenchyma. High-density iodinate oil can also be observed in the abscesses.

In the present study, 57.1% of the patients had a medical history of either bilioenteric anastomosis or biliary stent implantation. Hypoalbuminemia, portal vein cancer embolus, and diabetes mellitus were also found in 47.6%, 28.6%, and 23.8% of the patients, respectively. Moreover, particulate embolic agents were used in 42.9% of the patients. We speculate that these risk factors or comorbidities may be closely related to liver abscess formation, although the actual mechanism of abscess formation following TACE remains unclear. Further studies are therefore necessary to investigate the predisposing factors of liver abscess following TACE.

Microbiological cultures and antibiotics sensitivity test both have important values in selecting the appropriate antibiotics. In the present study, positive microbiological isolates were found in all pus cultures and in 10 blood cultures (47.6%). Gram-negative bacteria represented the largest proportion, in which *E coli* and *E cloacae* accounted for 52.4% and 38.1% of the total number of bacteria, respectively. In the previous investigation by Rahimian et al,^13^*E coli* accounted for 20.4% (11/54) of the microbiological isolates in pyogenic liver abscesses. However, in their series, all liver abscesses were not developed from TACE. Thus, whether or not the bacterial spectra of liver abscesses differ between patients who underwent TACE and those who did not remains to be further studied.

The literature on the management methods of liver abscess following TACE mainly comprises case reports and small case series. As such, the optimal treatment strategies for this type of abscess have long been a contentious issue.^14,15^ A meta-analysis revealed that liver abscesses ≤5 cm in diameter can undergo systemic antibiotic therapy, whereas larger (≥5 cm) or multiloculated abscesses and those that are associated with concomitant hepatobiliary and pancreatic diseases are recommended to undergo surgical drainage. PCD is recommended as a first-line treatment if no urgent surgical indications for peritonitis exist.^16^ In the present study, PCD was conducted on all types of abscesses; a 95.2% curative rate was obtained, without severe complications. Although all of the abscesses were larger than 5 cm in diameter, surgical drainage was not applied in our series because of the following major issues: the patients had a poor ECOG performance status score, which is frequently associated with hypoalbuminemia, liver and kidney dysfunction, and thrombocytopenia, such that they were intolerant of surgical drainage; surgical intervention may lead to tumor spread and metastases; the adhesion surrounding the liver caused by TACE may result in difficulty of surgical drainage.

The time course waiting for the results of microbiological cultures and antibiotic sensitivity tests is a key period for determining the patient's prognosis and outcome, which lasts about 5 to 7 days, because a fatal sepsis can occur during this period.^17^ Therefore, a broader spectrum and more aggressive antibiotic regimen should be empirically given before receiving the results of antibiotic sensitivity tests. The microbiological cultures in the present study suggested a wide variety of causative organisms, including gram negatives such as *E coli*, (52.4%, 11/21) and *E cloacae*, (38.1%, 8/21), as well as gram positives such as *E faecalis* (28.6%, 6/21) and *Staphylococcus epidermidis* (9.5%, 2/21), which is different from the earlier studies.^6,18^ A consensus on which antibiotic agent should be the first choice remains lacking to date. According to our bacterial spectrum, if liver abscesses following TACE are suspected, 3rd-generation cephalosporin and vancomycin should be used as the first-line antibiotics for obtaining a whole coverage of gram-positive and gram-negative organisms. When the results of antibiotic sensitivity tests were obtained, susceptible antibiotics can be chosen as the antibiotic regimen.

Liver abscess is a potentially fatal disease.^17–20^ Whether or not prophylactic antibiotics should be given during the peri-TACE period remains disputed, especially in patients who previously underwent bilioenteric anastomosis. Plentz et al^21^ compared 15 patients who received antibiotic prophylaxis with ciprofloxacin and metronidazole with those who received no prophylactic antibiotics. They found no difference in either the length of hospital stay or side effects between these patients. However, this study did not involve patients who have prior biliary reconstruction. Meanwhile, Kim et al^9^ applied prophylactic antibiotics in patients who had previously undergone Whipple procedures. A higher rate of subsequent liver abscess still developed among these patients. Geschwind et al^22^ suggested that bowel preparation in addition to prophylactic antibiotics is necessary to prevent abscess development. In our institution, 3rd-generation cephalosporin is intravenously infused 30 min before TACE. No additional antibiotics are used after then.

This study has several limitations. First, the drainage catheter is easily blocked by the sticky pus or the necrotic tissue debris. Therefore, a wide drainage catheter (14-Fr or 16-Fr in diameter) is recommended. Moreover, a thorough irrigation of abscess cavities with 0.9% saline is essential. Second, PCD is unsuitable for either immature or honeycomb-like abscesses. Fortunately, we did not encounter these types of abscess in our study. Third, PCD is inappropriate among patients with urgent conditions, such as peritonitis and abscess rupture. Finally, failure of PCD can lead to uncontrolled sepsis. Therefore, subsequent surgical interventions should be performed as soon as possible if PCD is unsuccessful.

In conclusion, liver abscess formation is an uncommon, but severe complication among patients who underwent TACE for liver malignancies. Patients with predisposing factors are prone to an increased risk. CT scans can reveal the characteristic features for the suspected patients. We explored an entire bacterial spectrum in a larger sample size compared with previous reports. The bacterial spectrum suggests that 3rd-generation cephalosporin and vancomycin should be used as the first-line antibiotics for obtaining a whole coverage of gram-positive and gram-negative organisms before the results of antibiotic sensitivity tests were obtained. PCD is a safe and effective method for patients with liver abscesses following TACE. Thus, PCD plus sensitive antibiotics can be recommended as the first-line treatment for these abscesses.

## References

[1] Han K Kim JH Yoon HM Transcatheter arterial chemoembolization for infiltrative hepatocellular carcinoma: clinical safety and efficacy and factors influencing patient survival. *Korean J Radiol* 2014; 15:464–471. 25053906 10.3348/kjr.2014.15.4.464 PMC4105809

[2] Zhou C Wang R Ding Y Prognostic factors for acute kidney injury following transarterial chemoembolization in patients with hepatocellular carcinoma. *Int J Clin Exp Pathol* 2014; 7:2579–2586. 24966972 PMC4069907

[3] Halpenny DF Torreggiani WC The infectious complications of interventional radiology based procedures in gastroenterology and hepatology. *J Gastrointestin Liver Dis* 2011; 20:71–75. 21451801

[4] Sun Z Li G Ai X Hepatic and biliary damage after transarterial chemoembolization for malignant hepatic tumors: incidence, diagnosis, treatment, outcome and mechanism. *Crit Rev Oncol Hematol* 2011; 79:164–174. 20719529 10.1016/j.critrevonc.2010.07.019

[5] Song SY Chung JW Han JK Liver abscess after transcatheter oily chemoembolization for hepatic tumors: incidence, predisposing factors, and clinical outcome. *J Vasc Interv Radiol* 2001; 12:313–320. 11287508 10.1016/s1051-0443(07)61910-1

[6] Chen C Chen PJ Yang PM Clinical and microbiological features of liver abscess after transarterial embolization for hepatocellular carcinoma. *Am J Gastroenterol* 1997; 92:2257–2259. 9399765

[7] Jianyong L Lunan Y Wentao W Barcelona clinic liver cancer stage B hepatocellular carcinoma: transarterial chemoembolization or hepatic resection? *Medicine (Baltimore)* 2014; 93:e180. 25474433 10.1097/MD.0000000000000180 PMC4616388

[8] Shibata T Yamamoto Y Yamamoto N Cholangitis and liver abscess after percutaneous ablation therapy for liver tumors: incidence and risk factors. *J Vasc Interv Radiol* 2003; 14:1535–1542. 14654488 10.1097/01.rvi.0000099532.29957.4f

[9] Kim W Clark T Baum R Risk factors for liver abscess formation after hepatic chemoembolization. *J Vasc Interv Radiol* 2001; 12:965–968. 11487677 10.1016/s1051-0443(07)61577-2

[10] Johnson GE Ingraham CR Nair AV Hepatic abscess complicating transarterial chemoembolization in a patient with liver metastases. *Semin Intervent Radiol* 2011; 28:193–197. 22654261 10.1055/s-0031-1280663 PMC3193326

[11] Kamran AU Liu Y Li FE Transcatheter arterial chemoembolization with gelatin sponge microparticles treated for BCLC stage B hepatocellular carcinoma: a single center retrospective study. *Medicine (Baltimore)* 2015; 94:e2154. 26717358 10.1097/MD.0000000000002154 PMC5291599

[12] Tu JF Huang XF Hu RY Comparison of laparoscopic and open surgery for pyogenic liver abscess with biliary pathology. *World J Gastroenterol* 2011; 17:4339–4343. 22090791 10.3748/wjg.v17.i38.4339 PMC3214710

[13] Rahimian J Wilson T Oram V Pyogenic liver abscess: recent trends in etiology and mortality. *Clin Infect Dis* 2004; 39:1654–1659. 15578367 10.1086/425616

[14] Chen SC Lee YT Lai KC Risk factors for developing metastatic infection from pyogenic liver abscesses. *Swiss Med Wkly* 2006; 136:119–126. 16633956 10.4414/smw.2006.11341

[15] Hope WW Vrochides DV Newcomb WL Optimal treatment of hepatic abscess. *Am Surg* 2008; 74:178–182. 18306874

[16] Chung YFA Tan YM Lui HF Management of pyogenic liver abscesses—percutaneous or open drainage? *Singapore Med J* 2007; 48:1158–1165. 18043848

[17] Ryan JM Ryan BM Smith TP Antibiotic prophylaxis in interventional radiology. *J Vasc Interv Radiol* 2004; 15:547–556. 15178714 10.1097/01.rvi.000024942.58200.5e

[18] Ong GY Changchien CS Lee CM Liver abscess complicating transcatheter arterial embolization: a rare but serious complication. A retrospective study after 3878 procedures. *Eur J Gastroenterol Hepatol* 2004; 16:737–742. 15256974 10.1097/01.meg.0000108361.41221.8c

[19] Vanderwalde AM Marx H Leong L Liver abscess as a complication of hepatic transarterial chemoembolization: a case report, literature review, and clinical recommendations. *Gastrointest Cancer Res* 2009; 3:247–251. 21151429 PMC3000072

[20] Leiding JW Freeman AF Marciano BE Corticosteroid therapy for liver abscess in chronic granulomatous disease. *Clin Infect Dis* 2012; 54:694–700. 22157170 10.1093/cid/cir896 PMC3275758

[21] Plentz RR Lankisch TO Bastürk M Prospective analysis of German patients with hepatocellular carcinoma undergoing transcatheter arterial chemoembolization with or without prophylactic antibiotic therapy. *J Gastroenterol Hepatol* 2005; 20:1134–1136. 15955232 10.1111/j.1440-1746.2005.03836.x

[22] Geschwind JF Kaushik S Ramsey DE Influence of a new prophylactic antibiotic therapy on the incidence of liver abscesses after chemoembolization treatment of liver tumors. *J Vasc Interv Radiol* 2002; 13:1163–1166. 12427817 10.1016/s1051-0443(07)61959-9

